# Supplementary material for: Nutritional Content of Non-Dairy Frozen Desserts
Source: Nutrients. 2022 Oct 6;14(19):4150. doi: 10.3390/nu14194150 (PMC9571912; doi:10.3390/nu14194150)
Supplement: Supplementary file 1 [file nutrients-14-04150-s001.zip › nutrients-1885944-supplementary.pdf]

Supplement S1. Details on the location and collection of frozen dessert samples, in addition to that stated in the Materials and Methods Section.

Products were identified by looking in the non-dairy section next to the dairy products of supermarkets up and down the west coast. The senior author took photos of the nutrition label to record the data at home. Products analyzed had to be labeled as non-dairy plant based. Supermarkets were located in Spokane and Walla Walla, Eastern Washington; Bend, Oregon; Boise, Idaho; Salt Lake City and Moab, Utah; San Jose, Loma Linda, Redlands, Grand Terrace, California; Los Angeles county, and Riverside county, California were searched for products. This included health food stores in those towns, and included main supermarket chains such as Walmart, Aldi's, Sprouts, Safeway, Albertsons, Whole Foods Markets, Trader Joe's, Stater Brothers, and Grocery Outlet. Smaller health food stores were also covered including Andy's and Super 1 in Walla Walla, WA; Loma Linda Market, and Clarks Nutrition and Natural Foods Market in Loma Linda, CA. Data collection took place over the first 4 months of 2022.

The website https://www.godairyfree.org/product-reviews/frozen-desserts lists product reviews of all non-dairy beverages, creamers, yogurts, cheeses and frozen desserts etc. sold in the USA. From this comprehensive and up-to-date web site one can get a list of other brands that are available in the US, other than from the Western states. This leads you to the manufacturers website where you can find all the varieties they presently sell with the nutrition information for each product. Only those products with incomplete nutritional information on the web (very few) were excluded from the analysis.

Table S2. Statistical analysis results from Kruskal–Wallis non-parametric tests used for each nutrient to perform comparisons among base types for ice cream pints.

Nutrient	Chi squared value	df	p-value
Calories	97.83	16	8.84E-14
Fat	76.75	16	6.37E-10
Saturated Fat	173.55	16	<2.2E16
Sodium	94.8	16	3.24E-13
Carbohydrate	154.09	16	<2.2E-16
Fiber	102.28	16	1.29E-14
Total Sugar	125.26	16	<2.2E-16
Protein	113.94	16	<2.2E-16

Table S3. Statistical analysis results from Kruskal–Wallis non-parametric tests used for each nutrient to perform comparisons among base types for ice cream bars and sandwiches.

**Nutrient**	**Chi squared value**	**df**	**p-value**
Calories	3.48	4	0.482
Fat	7.43	4	0.115
Saturated Fat	14.56	4	0.005
Sodium	6.86	4	0.143
Carbohydrate	6.48	4	0.481
Fiber	12.28	4	0.015
Total Sugar	3.09	4	0.0543
Protein	7.00	4	0.136

Table S4. Medians of calories and 3 nutrients in dairy ice cream and dairy-free frozen desserts[1].

	N	Calories	Saturated fat	Added sugars	Protein
Dairy Ice Cream	45	180	4.5	14	4
Dairy-Free Frozen Desserts	14	225	8.5	19	2

[1]Analysis performed on the data given in reference 25.
